# Production of transgenic pigs over-expressing the antiviral gene Mx1

**DOI:** 10.1186/2045-9769-3-11

**Published:** 2014-09-26

**Authors:** Quanmei Yan, Huaqiang Yang, Dongshan Yang, Bentian Zhao, Zhen Ouyang, Zhaoming Liu, Nana Fan, Hongsheng Ouyang, Weiwang Gu, Liangxue Lai

**Affiliations:** 1 Guangzhou Institutes of Biomedicine and Health, Chinese Academy of Sciences, Guangzhou, China; 2 College of Animal Sciences, Jilin University, Changchun, China; 3 Institute of Comparative Medicine and Center of Laboratory Animals, Southern Medical University, Guangzhou, China

**Keywords:** Antiviral breeding, Innate resistance, Somatic cell nuclear transfer

## Abstract

The myxovirus resistance gene (Mx1) has a broad spectrum of antiviral activities. It is therefore an interesting candidate gene to improve disease resistance in farm animals. In this study, we report the use of somatic cell nuclear transfer (SCNT) to produce transgenic pigs over-expressing the Mx1 gene. These transgenic pigs express approximately 15–25 times more Mx1 mRNA than non-transgenic pigs, and the protein level of Mx1 was also markedly enhanced. We challenged fibroblast cells isolated from the ear skin of transgenic and control pigs with influenza A virus and classical swine fever virus (CFSV). Indirect immunofluorescence assay (IFA) revealed a profound decrease of influenza A proliferation in Mx1 transgenic cells. Growth kinetics showed an approximately 10-fold reduction of viral copies in the transgenic cells compared to non-transgenic controls. Additionally, we found that the Mx1 transgenic cells were more resistant to CSFV infection in comparison to non-transgenic cells. These results demonstrate that the Mx1 transgene can protect against viral infection in cells of transgenic pigs and indicate that the Mx1 transgene can be harnessed to develop disease-resistant pigs.

## Introduction

Type I interferons (IFNs) are important mediators of the innate immune responses and are crucial for limiting the early replication and spread of viruses [1]. An important downstream effector of type I IFNs is the myxovirus resistance gene (Mx1 in mice and pigs, MxA in humans). The expression of Mx1 is strongly induced by IFN-α/β,double-stranded RNA or viral infections [2, 3]. The Mx1 protein can protect numerous hosts by preventing the growth of a wide variety of viruses both in vitro and in vivo including orthomyxoviruses [4–6], bunyaviruses [7, 8], rhabdoviruses [9], paramyxoviruses [10] and hantaviruses [11, 12]. Several studies showed that stably transfected cells constitutively expressing chicken, mouse, pig or human Mx1/MxA proteins effectively inhibited the replication of several RNA viruses [13–19]. Specifically, transgenic mice expressing mouse Mx1 or human MxA also showed enhanced resistance to the influenza A virus as well as to several other viruses [20, 21]. Collectively, these studies showed that the resistance of transgenic mice to viral infection was not due to broad immunological activation in response to the virus. Rather, Mx1/MxA proteins exert their antiviral effects by directly inhibiting replication of the viral genome.

Despite the potent antiviral activity of Mx1, its utility to protect large animals, such as pigs, from viral infection has not yet been explored. Pigs are susceptible to swine, human and avian influenza viruses. As alternate hosts, pigs are believed to be “mixing vessels” by enabling the genetic rearrangement of influenza viruses and thereby amplify their genetic variation. As a consequence, the probability of influenza pandemics threatening humans is increased [22]. A recent report revealed that the circulating pandemic H1N1/2009 influenza A virus originated in pigs and contained genetic contributions of human, avian and swine influenza viruses [23]. Therefore, influenza resistant pigs would not only benefit the livestock industry but also global public health by eliminating a critical host so that new variants of pandemic influenza strains can no longer emerge. Mx1 is an interesting candidate gene to engineer pigs with enhanced viral resistance. Besides influenza, Mx1 is also expected to lead to resistance for other severe swine infectious viruses such as CSFV. As the use of swine vaccines to prevent these diseases is very costly, the production of genetically modified pigs that possess congenital resistance to viral infection is an attractive avenue to explore.

In this study we tested the hypothesis that Mx1 could be used to generate pigs that resist viral infection. Previously, Mx1 transgenic pigs had been generated using pronuclear injection. However, these animals failed to express exogenous Mx1 protein [24]. SCNT technology enables the efficient generation of genetically modified large animals. The successful cloning and production of genetically modified pigs by transferring nuclei of transgenic or gene targeted somatic cells have previously been reported [25–27]. In this study, we produced transgenic pigs over-expressing the Mx1 gene using the SCNT technology and explored its potency to protect porcine cells from viral infection.

## Materials and Methods

### Vector construction and selection of transgenic donor cells

Fetal fibroblasts derived from Tibetan miniature pigs were cultured in Dulbecco’s modified Eagle’s medium (DMEM, HyClone, Logan, UT, USA) supplemented with 15% fetal bovine serum (FBS, HyClone, Logan, UT, USA) and 1% (v:v) penicillin/streptomycin (10,000 U/ml penicillin, 10,000 μg/ml streptomycin; GIBCO-BRL, Grand Island, NY, USA) at 39°C in an incubator with 5% CO_2_. Cultured fibroblasts were stimulated with 1000 U/ml IFN-α (PeproTech, Rocky Hill, NJ, USA) for 6 hours. Total RNA was extracted with TRIzol (Invitrogen, Carlsbad, CA, USA) as described by the manufacturer. First strand cDNA was synthesized by M-MLV Reverse Transcriptase (Takara, Dalian, China). The following primers were used to amplify the pig Mx1 gene by PCR based on reported DNA sequence (gene ID: 397128): Mx1-F: 5’-TAATCTAGAATGGTTTATTCCAGCTGTGAAAGTAAAG-3’, Mx1-R: 5’-GCCAAGCTTGCCTGGGAACTTGGCGAGCC-3’. The PCR product was digested using Xba I/Hind III and ligated to the plasmid pBS-2A-enhanced green fluorescent protein (EGFP) constructed by our lab to form the transgenic vector pMx1-2A-EGFP. In addition, the Mx1 gene was cloned into the pVAX1 (Invitrogen, Carlsbad, CA, USA) to construct the pVAX-Mx1 vector lacking the fusion protein. This vector was used as a positive control in identifying the expressed protein products of transgenic vector. The final constructs were confirmed by sequencing. To test whether 2A could be used to discriminate Mx1 and Mx1-EGFP proteins, 4 μg pMx1-2A-EGFP, pVAX-Mx1 and empty vectors (pBS-2A-EGFP and pVAX1) were transfected into the 293 T cells (10^6^) using Lipofectamine 2000 (Invitrogen, Carlsbad, CA, USA). Cells were harvested and lysed 48 hours later. Equal amounts of total proteins were separated on a 10% polyacrylamide gel and transferred to PVDF membrane (Millipore, Bedford, MA, USA). The proteins were detected with mouse anti-Mx1 (1:500 dilution, ab79609; Abcam, Cambridge, UK) and mouse anti-GAPDH (1:2000 dilution, 60004-1-Ig; Proteintech, Chicago, IL, USA) antibodies. A horseradish peroxidase conjugated goat anti-mouse IgG secondary antibody (1:2000 dilution, sc-2005; Santa Cruz, CA, USA) and the ECL Plus detection system (Amersham Pharmacia Biotech, Arlington Heights, IL, USA) was used for visualization.

Primary pig fetal fibroblasts were isolated from a 25 day-fetus of a female Tibetan miniature pig. pMx1-2A-EGFP was linearized with ApaLI and transfected into the fibroblasts by electroporation (Gene Pulser Xcell, Bio-rad, Hercules, CA, USA). The cells were split 1:10 into fresh culture medium after transfection. After 48 hours, 1000 μg/ml G418 (Merck, Darmstadt, Germany) was added to the medium to select transgenic cell colonies for approximately 2 weeks. The surviving cell colonies that expressed EGFP were selected and propagated in a fresh 48-well plate. Colonies that proliferated well were then expanded and screened for the presence of the transgene. The positive colonies were frozen in small aliquots. Prior to SCNT, Mx1-expressing transgenic cells were thawed and cultured until they reached sub-confluence.

### Production of Mx1 transgenic pigs

SCNT was performed as described [26]. The reconstructed embryos were then surgically transferred into the oviduct of a surrogate female on the first day of standing estrus. The pregnancy status was monitored using an ultrasound scanner between 30–35 days post-transplantation. Some embryos were cultured for 6 days to test the blastocyst formation rate as well as developmental ability.

### Identification of transgenic pigs

**Figure 1:**
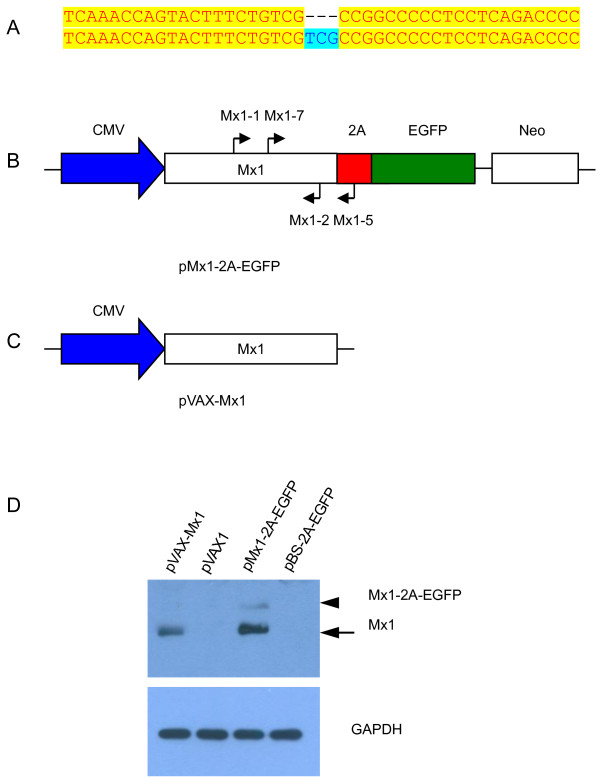
**Construction and expression of Mx1 transgenic vector. (A)** Comparison of 2 alleles of Mx1 gene in Tibet miniature pigs (position 1675–1718 in nucleic acid sequence). The 3 base pair (bp)-deleted sequence is indicated with a dashed line. **(B)** Schematic diagram of the transgenic vector pMx1-2A-EGFP and the binding sites of primers used in genomic PCR assays to screen for the presence of the transgene are marked with arrows. The size of the PCR product using primers Mx1-2 and Mx1-7 is 192 bp. The same primers generate a PCR fragment of 575 bp from the endogenous genomic Mx1 due to the existence of an intron. The primer Mx1-5 is located in the 2A sequence and the transgenic vector produces a 750 bp DNA fragment when amplified by PCR using primers Mx1-1 and Mx1-5. These primers cannot amplify wild-type genomic DNA. **(C)** Schematic diagram of the Mx1 expression vector pVAX-Mx1. **(D)** Transient expression of pMx1-2A-EGFP and pVAX-Mx1 in 293 T cell. The arrowhead indicates the uncleaved transgenic Mx1-2A-EGFP protein. Arrow indicates the cleaved Mx1 protein.

Genomic DNA was extracted from the ear tissues of newborn cloned pigs for PCR analysis. The following primers were used to amplify the Mx1 gene fragments: Mx1-1: 5’- CAAATGGAGTGCTGTGGTTG-3’, Mx1-2: 5’-GCAGTACACGATCTCCA-3’, Mx1-5: 5’- ACAGGAGCGACAATTTTAAGC-3’ and Mx1-7: 5’- CGCCTTCACAGATGTTTCAG-3’. The binding sites of these primers are shown in Figure 1B. The expected sizes of PCR products of the transgenic Mx1 and endogenous Mx1 genes differ due to the presence of introns in the endogenous Mx1. Total RNA was extracted from fibroblasts isolated from the ear tissues of newborn cloned piglets using the RNeasy Mini Kit (Qiagen, Valencia, CA, USA), subjected to reverse transcription (PrimeScript RT Master Mix, Takara, Dalian, China) and real time RT-PCR (SYBR Premix Ex Taq™, Takara, Dalian, China) to determine the expression levels of Mx1 mRNA. The expression values for the Mx1 mRNA were normalized to the expression values obtained for GAPDH. The following primers were used for real time RT-PCR: Mx1-q1: 5’-CACAGAACTGCCAAGTCCAA-3’, Mx1-q2: 5’-GCAGTACACGATCTGCTCCA-3’, GAPDH-q1: 5’-CAGCAATGCCTCCTGTACCA-3’ and GAPDH-q2: 5’-GATGCCGAAGTTGTCATGGA-3’. RNA samples from 3 newborn natural breeding Tibet miniature piglets were used as controls. Western blot were used to detect the expression of Mx1 protein in the transgenic pigs as described above using fibroblast lysates and organ lysates of transgenic pig and non-transgenic pig control.

### Determination of transgene copy numbers

Real time RT-PCR was performed to determine the integrated transgene copy number by means of SYBR^®^ Premix Ex Taq™ II (Takara, Dalian, China). Genomic DNA of transgenic and wild-type pigs was used in duplicate in a 20 μl reaction using CFX96 Touch™ Real-Time PCR Detection Sysctem (Bio-Rad, Hercules, USA). Primer sequences were designed as follow: gMx1-1: 5’-CCACATCCCTCTGATCATCC-3’, gMx1-2: 5’-CAGGAGCCAGTCGTATTGGT-3’, gGAPDH-1: 5’-CTTTGCCCCGCGATCTAATG-3’ and gGAPDH-2: 5’-CTCACCCGTTCACTCCGACC-3’. Mx1 primers were designed to amplify the same sequences in transgenic Mx1 and endogenous genomic Mx1. Transgene copy number per individual was calculated by normalization to GAPDH. Since wild type pig has 2 copies of Mx1, transgene copy number of transgenic pigs can be calculated based on the relative value to the wild type pigs.

### Isolation of cells from the transgenic pigs

Pig fibroblast cells were isolated from ear tissues of transgenic and age-matched non-transgenic pigs. Tissues were washed twice with PBS and finely chopped (1–2 mm^3^) using scissors. Tissue pieces were then suspended in DMEM supplemented with 15% FBS and 5% (v/v) penicillin/streptomycin at 39°C in an incubator with 5% CO_2_. Cells migrated from the tissue pieces onto the surface of the tissue culture dishes during the next few days. The floating pieces of tissues were removed by aspiration on day 5–6 of culture. When confluent, monolayers of fibroblast-like cells were harvested with trypsin and passaged in the medium described above.

### Infection of transgenic fibroblasts with influenza A virus

Influenza A (H1N1) virus strain A/Puerto Rico/8/34 (PR8) and H5N1 recombined strain NIBRG-14 (A/Vietnam/1194/2004), were propagated in 7 day-old embryonated chicken eggs. The allantoic fluid containing the virus was collected and the viral titer was determined by a plaque assay [28]. Fibroblasts from the Mx1 transgenic pigs and non-transgenic controls were cultured in 24-well plates. When confluent, cell monolayers were infected with influenza A virus at a multiplicity of infection (MOI) of 1 plaque-forming unit (pfu) per cell. One hour post-infection, non-internalized virus was discarded by removal of the supernatant and cell monolayers were washed thoroughly with PBS. Then fresh medium (DMEM containing 0.3% BSA and 0.5 μg/ml TPCK-trypsin, Fluka, Buchs, Switzerland) was added to the infected monolayers. 24 hours later, infected cells were fixed in 4% paraformaldehyde containing 0.1% Triton X-100 and then labeled with mouse anti-influenza A nucleoprotein (NP) antibody (1:100 dilution, sc-80481; Santa Cruz, CA, USA) and stained with Alexa Fluor 568-conjugated goat anti-mouse IgG secondary antibody (1:200 dilution). After washing in PBS, the stained cells were examined by an inverted fluorescence microscope. To quantify the number of positive cells, cells were counted in more than 3 sections for each sample using 10× microscopic fields to determine the percentage of positive cells.

To accurately determine viral copies in the infected cells, fibroblasts cultured in 24-well plates were infected with influenza A virus PR8 and NIBRG-14 at an MOI of 1 and 0.01 pfu per cell, respectively (3 replicate wells for each sample). We collected the supernatant of cultured cells at 0, 5, 10, 15, 20, and 25 hours post-infection, extracted viral RNA from 200 μl of the cell culture supernatant of each sample, and performed real time PCR to identify the number of copies of the NP gene in the supernatant. To generate a standard curve, the viral NP gene was cloned into the pGEM-T vector (Promega, Madison, WI, USA) and serially diluted to generate samples containing 1-10^8^ copies per ml of the plasmid. Viral titers were calculated as the number of copies of the NP gene per ml of viral supernatant and subsequently converted to their log values using the standard curve [29]. The primer Uni12, 5’-AGCAAAAGCAGG-3’ was used to generate cDNA of all genes of the influenza A virus; and primers NP-F, 5’-TGTATGGACCTGCCGTAGC-3’ and NP-R, 5’-CCCTCTTGGGAGCACCTT-3’ were used to amplify the NP gene.

### Infection of transgenic fibroblasts with CSFV

The highly virulent CSFV Shimen strain was obtained from the Institute of Veterinary Drug Control, China. Positive anti-CSFV serum and negative control serum were prepared as described previously [30]. Freshly trypsinized fibroblasts of the Mx1 transgenic pigs and age-matched controls were added to 24-well plates and incubated at 39°C in 5% CO_2_. When 80-90% confluent, cells were infected with the CSFV Shimen strain (400 TCID_50_ per well). At 60 hours post infection, the culture media were aspirated and the cell monolayers in wells were fixed with 80% cold acetone for 30 minutes, washed 3 times with PBS, and incubated for 1 h with pig anti-CSFV serum (1:100 dilution) at 37°C in a humidified box. Cells were then washed 3 times with PBS and incubated with FITC-conjugated rabbit anti-pig IgG (1:60 dilution, F4762; Sigma) for 1 hour. After thoroughly washing, positive cells were photographed and counted under an inverted fluorescence microscope. The FITC fluorescence of the secondary antibody stain could be observed clearly using a 4 × objective lens and there was no interference of the EGFP signal in the transgenic cells at this amplification level.

## Results

### Generation of transgenic pigs over-expressing Mx1

We detected 2 isoforms of Mx1 mRNA in Tibet miniature pigs. This is in accordance with previous studies reporting a polymorphism in exon 13 of the porcine Mx1 gene [18]. One of the alleles has a 3 bp-deletion at position 1696–1698 leading to the elimination of a serine residue at position 565 (Figure 1A). Both Mx1 isoforms were reported to possess indistinguishable antiviral activities [18]. Previous reports also described a third isoform of porcine Mx1 characterized by an 11-bp deletion in exon 14 causing a frameshift mutation in the carboxyl terminal region of the Mx1 protein leading to an impairment of its antiviral activities [18, 31, 32]. However, we did not detect this isoform in the Tibet miniature pigs. Here we decided to use the longest Mx1 isoform to construct transgenic vectors. The plasmid was constructed by fusing the porcine Mx1 gene to an EGFP gene separate by a 2A peptide linker (Figure 1B). EGFP was used as a reporter to readily identify transgenic cells and pigs. The 2A peptide can mediate self-cleavage of the fusion protein via a ribosomal skipping mechanism [33]. However, as this peptide is short, it exerts no adverse effects on the structure and function of either the upstream or downstream proteins [34, 35]. Further, we constructed a vector that expressed Mx1 without an EGFP fusion as a control for testing the Mx1 expression in vitro (Figure 1C). Transient transfection of HEK293T cells showed that the majority of the Mx1 could be efficiently separated from EGFP (Figure 1D). However, a small amount of uncleaved fusion protein was still detectable.

After transfection and selection, we selected three individual fetal fibroblast cell lines that expressed the Mx1 transgene as nuclear donors and transferred a total of 2283 SCNT embryos into 14 surrogate mothers that exhibited natural estrus. Seven surrogates developed to term and gave birth to 18 female piglets after 120-130 days of gestation. One piglet died at birth and two died 2 days after birth. A fourth piglet (#4-4 transgenic pig) suffered a spine injury caused by the surrogate mother during lactation. It died because of reduced body weight gain at about 6 months of age. Fourteen piglets survived to date (Table 1). Most live piglets appeared normal at birth and there was no obvious difference in appearance between the cloned and natural bred piglets.

**Figure 2:**
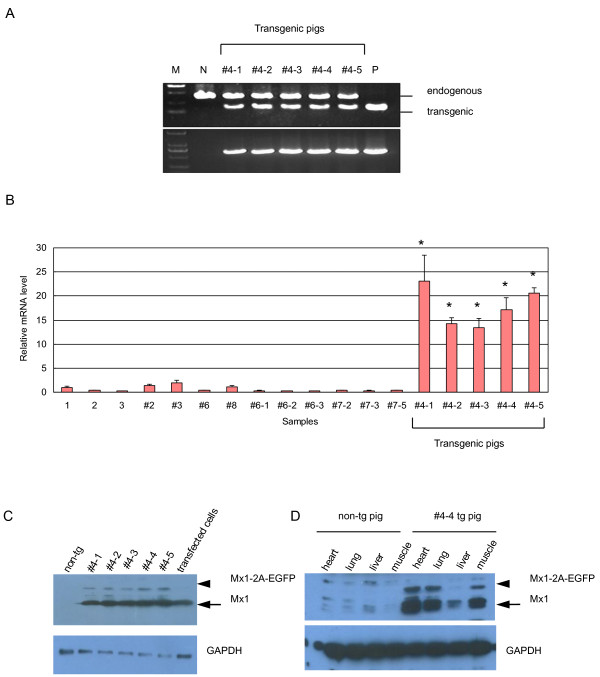
**Genotyping and expression analysis of Mx1 transgene in trangenic pigs. (A)** PCR to identify piglets with genomic integration of the Mx1 transgene. Upper panel: PCR analysis with primers Mx1-2 and Mx1-7; Lower panel: PCR analysis with primers Mx1-1 and Mx1-5. The lanes are: M, marker; N, non-transgenic pig; #4-1, #4-2, #4-3, #4-4, #4-5, transgenic pigs; P, plasmid DNA. **(B)** Identification of mRNA level of Mx1 in the transgenic piglets using real time RT-PCR. Lanes 1–3 are naturally bred piglets, lanes 4–13 are cloned pigs without transgene integration, and lanes 14–18 are 5 cloned pigs that contained the transgene. Porcine GAPDH was used as reference control. Values represent the mean ± s.d. from triplicate experiments. Statistically significant P values are noted with an asterisk (*P < 0.001. One-way natural breeding pig was used to generate the P values). **(C)** Detecting the presence of Mx1 protein in fibroblasts of transgenic pigs by western blot. Cell lysates from HEK293T cells transiently transfected with pVAX-Mx1 were used as a positive control. **(D)** Expression of the Mx1 protein in various organs of transgenic and non-transgenic pigs. The arrowhead indicates the uncleaved transgenic Mx1-2A-EGFP protein and the arrow indicates the cleaved Mx1 protein.

**Figure 3:**
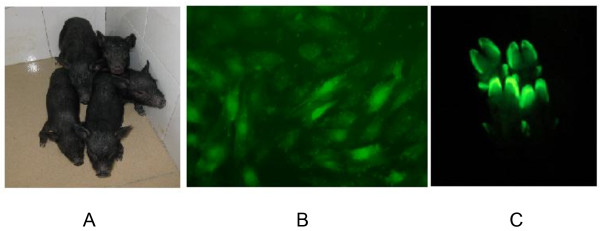
**Pictures of transgenic pigs and EGFP expression in the fibroblasts and hooves. (A)** Picture of the 5 transgenic piglets taken at 1 month of age. **(B)** Expression of EGFP in ear fibroblasts isolated from a transgenic piglet; 20 × magnification. **(C)** Expression of EGFP in the hooves of a transgenic piglet.

PCR analysis of genomic DNA of each piglet showed that 5 piglets were positive for the Mx1 transgene and all of which had been derived from transgenic cell colony #4 (Figure 2A). Real time RT-PCR analysis revealed that these transgenic piglets carried 5 copies of the transgene. Additionally, we determined that Mx1 mRNA levels in fibroblasts isolated from the 5 transgenic piglets were 15–25 times higher than in cloned piglets lacking the transgene as well as in naturally bred piglets (Figure 2B). Western blot showed enhanced Mx1 protein levels in fibroblasts isolated from transgenic pigs (Figure 2C). Mx1 protein levels were also elevated in the organs (heart, lung, liver, muscle) of the deceased transgenic pig #4-4 as compared to non-transgenic pigs (Figure 2D). We also observed EGFP expression in fibroblasts and hooves of all 5 transgenic piglets further illustrating the successful generation of transgenic animals that robustly express the Mx1 gene in a wide variety of tissues (Figure 3A, B, C).

**Table 1:** **Summary of SCNT results for the generation of** ***Mx1*** **transgenic pigs**

Nuclear donor cell lines	#1	#2	#3
Embryos transferred to recipients	937	855	491
Recipients	6	5	3
Pregnancies	2	4	1
Pregnancies brought to term	2	4	1
Born piglets	7	6	5
Live piglets	5	5	4
Transgenic piglets	0	0	5

### Fibroblasts isolated from Mx1 transgenic pigs are more resistant to influenza A viral infection

**Figure 4:**
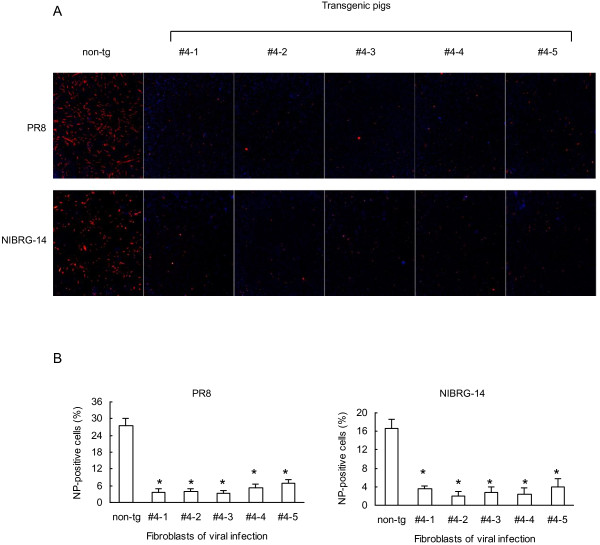
**Enhanced influenza A viral resistance in cultured fibroblasts isolated from transgenic pigs. (A)** Ear fibroblasts isolated from Mx1 transgenic pigs and age-matched controls were infected with influenza A virus PR8 or NIBRG-14. After 24 hours, cells were fixed and stained for NP expression (red: NP, blue: nuclei; 4 × magnification). **(B)** Quantification of NP-positive cells. Values represent the mean ± s.d., n = 3. *P < 0.01 compared with the non-transgenic group.

**Figure 5:**
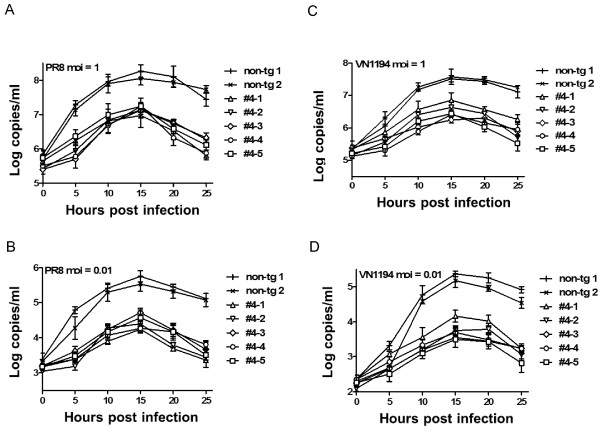
**Growth curves of influenza A viruses PR8 and NIBRG-14 in the isolated ear fibroblasts of Mx1 transgenic piglets.** Growth curves of PR8 at a MOI of 1 **(A)** and 0.01 **(B)**. Growth curves of NIBRG-14 at a MOI of 1 **(C)** and 0.01 **(D)**. Values represent the mean ± s.d., n = 3.

Next we asked whether high expression levels of Mx1 rendered transgenic cells resistant to influenza A infection. To test this proposition, ear fibroblasts from transgenic pigs as well as non-transgenic controls were isolated and challenged with two different strains of influenza A viruses, PR8 and NIBRG-14. After 24 hours, we monitored infection by influenza A viruses using the IFA assay. We observed that the replication of the 2 influenza A strains was profoundly decreased in fibroblasts transgenic for Mx1 (Figure 4). Next, we monitored the viral growth curve by real time RT-PCR (Figure 5). When challenged with PR8 virus at an MOI of 1, we observed substantially lowered titers of PR8 virus in the transgenic versus the non-transgenic cells (Figure 5A). When the viral titers peaked at 15 hours post infection, there were approximately 10-fold less viral copies in the transgenic cells than in the non-transgenic cells. Following the peak at 15 h, viral titers began to decline, and this process occurred more rapidly in the Mx1 transgenic cells than in the non-transgenic controls. At 20 and 25 h post-infection, there was a 10^1.5^ to 10^2^-fold difference in the number of viral copies between the transgenic cells and the non-transgenic cells (Figure 5A, B). Although cells infected at an MOI of 0.01 produced overall lower viral titers than at an MOI of 1, they exhibited similar viral replication profiles and reduced viral copies in transgenic as compared to control cells. Similar differences in the kinetics of infection were observed between transgenic and non-transgenic cells when the NIBRG-14 influenza strain was used (Figure 5C, D).

### Fibroblasts isolated from Mx1 transgenic pigs are more resistant to CSFV infection

**Figure 6:**
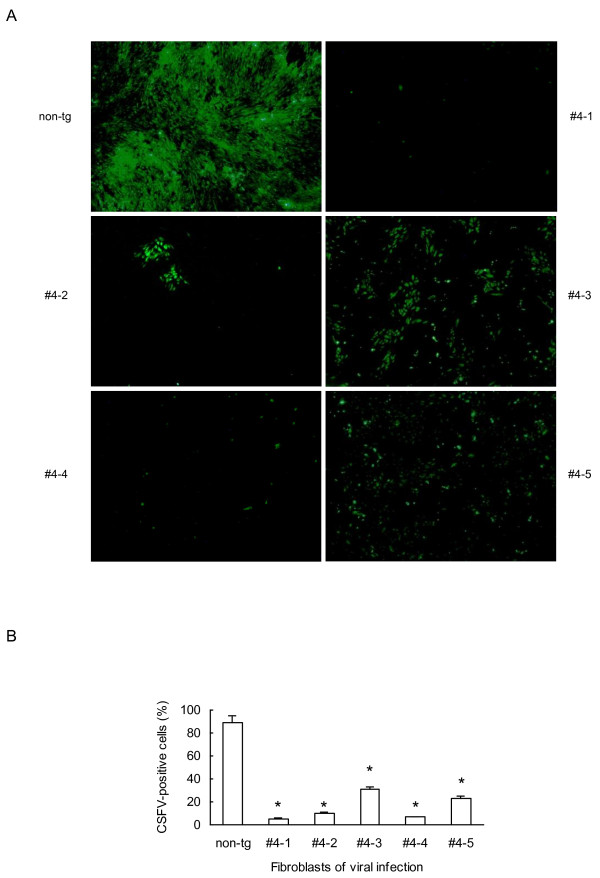
**Protective effect of Mx1 transgene against CSFV infection. (A)** IFA and FITC conjugated antibodies were used to examine the viral infection in fibroblasts from 5 transgenic and an age-matched non-transgenic pigs; 4 × magnification. **(B)** Quantification of CSFV-positive cells. Values represent the mean ± s.d., n = 3. *P < 0.01 vs non-transgenic.

Finally we wanted to study the inhibitory effects of the Mx1 transgene on a virus other than influenza. To this end, we infected fibroblast cells with CSFV and examined its replication by detecting levels of the virus by IFA using anti-CSFV serum. At 60 hours post-CSFV infection, the fibroblasts from non-transgenic controls exhibited bright green fluorescence in the cytoplasm, indicating that most cells were producing the virus (Figure 6). By contrast, fewer cells from Mx1 transgenic pigs displayed green fluorescence. We observed some variation between cells derived from different pigs. Cells from pigs #4-1, #4-2, #4-4 displayed the lowest degree of green fluorescence, indicating that the inhibitory effects on CSFV replication is strongest in these cells (Figure 6).

## Discussion

Pigs are natural hosts for influenza A, CSFV and several other viruses. To combat viral infections, the porcine Mx1 gene has co-evolved with these viruses for thousands of years to inhibit the viral life cycle. Even a virus with tremendous genetic plasticity cannot durably bypass this innate resistance mechanism. All vertebrates possess between 2 and 3 IFN-inducible Mx-like genes. Mx proteins from different species target alternative viruses, illustrating the functional diversity of this protein family and its adaptability to combat viral infections [36]. It has been reported that the pig Mx1 inhibits the replication of the influenza A virus by blocking the endocytic cascade of invading influenza A particles [37]. The pig Mx1 protein is rapidly found in the cytoplasm in response to acute viral infections [38–40]. Cells adjacent to infected tissues also express Mx1 in vivo. Here we hypothesized, that if Mx1 would be expressed constitutively and ubiquitously in all porcine cells, animals would be armed to promptly combat and thus resist viral infections. Indeed, Su *et al.* [41] produced transgenic fish over-expressing the fish Mx1 gene (GrMx), and these transgenic fishes exhibited increased resistance to grass carp reovirus infection.

There is ample evidence, both *in vivo* and *in vitro*, that pigs serve as facile hosts for the genetic reassortment of influenza A viruses facilitating the evolution of genetically novel viral strains [42–44]. These “new” influenza strains can be generated in pigs that have been co-infected with human, avian, and swine viruses. This phenomenon has not only been confirmed by phylogenetic and epidemiologic analyses, but also by molecular studies that identified hybrid viruses arising through genetic recombination of diverse parental viruses within the porcine host cells. Although influenza A viruses preferentially recognize cell surface oligosaccharides with a terminal sialic acid, alternative entry routes do also exist. Most avian influenza strains preferentially bind to the N-acetylneuraminic acid-α2,3-galactose (NeuAcα2,3Gal) linkage on sialyloligosaccharides, while human influenza strains prefer the NeuAcα2,6Gal linkage. Cells of the upper respiratory tract in pigs often contain both NeuAcα2,6Gal and NeuAcα2,3Gal sialyloligosaccharides [22]. This provides an environment that is conducive to co-infection with human and avian influenza viruses and thus favors the emergence of variant, potentially pandemic influenza A strains. Additionally, 1 report has indicated that avian viruses that infected pigs but failed to replicate could still contribute genetically to newly evolving viral strains [44]. Collectively, pigs afford many features that favor the “mixing” of a variety of influenza strains, designating the pig an alternate host that facilitates the dissemination and evolution of the influenza A virus.

Additionally, the CSFV leads to a highly contagious disease of pigs. Outbreaks of CSFV infections usually leads to significant economic losses in many countries worldwide. Considering the broad-spectrum antiviral activities of Mx1, we set out to test whether this gene could confer multi-virus resistance in pigs and also prevent CSFV infections. The inhibitory activity of Mx1 on CSFV had previously not been assessed.

In our experiments, we fused the Mx1 transgene to the EGFP reporter to construct a transgenic vector in which a 2A peptide could be used to discriminate between different fusions and endogenous Mx1. EGFP was useful to facilitate the selection of transgenic cell colonies and to conduct the nuclear transfer. Transient expression of transgenic vector in HEK293T cells showed that most of the fusion protein could be efficiently cleaved, which encouraged us to produce transgenic pigs using this vector. When the Mx1 transgenic pigs were born, we used fibroblasts isolated from the ear tissues to evaluate the antiviral activities of the Mx1 transgene in the pigs. Ideally, epithelial cell from the respiratory tract should be used to evaluate the protection from viral infection in transgenic pigs. However, such cells are hard to obtain without sacrificing the animal. We could detect higher Mx1 expression levels in isolated fibroblasts at both mRNA and protein levels. Encouragingly, we observed an enhanced resistance to influenza A virus and CFSV infection in the fibroblasts isolated from the Mx1 transgenic pigs as compared to non-transgenic pigs. Overall, the influenza A virus replicates with only moderate efficiency in pig fibroblasts as compared to other cell types (28% infected cells for PR8 and 16% infected cells for NIBRG-14). Nevertheless, the presence of the Mx1 transgene led to a further repression of the viral replication.

Collectively, we report for the first time the production of transgenic pigs over-expressing the antiviral gene Mx1. Clearly, further studies are needed to comprehensively elucidate the antiviral activities of Mx1 transgenic pigs. Nevertheless, the use of Mx1 transgenic pigs offers a novel approach to explore avenues to prevent and control the evolution and the spread of pandemic influenza strains or CSFV. The broad-spectrum antiviral properties of the Mx1 gene may also provide resistance to other swine infectious diseases, such as the porcine respiratory and reproductive syndrome and foot-and-mouth disease. The transgenic pigs produced in this study allow the testing of a variety of swine viruses. These experiments would show if transgenic Mx1 confers resistance to these viruses and reveals the utility of these transgenic animals for the betterment of global public health.

## Abbreviations

CFSV: Classical swine fever virus
EGFP: Enhanced green fluorescence protein
IFA: Indirect immunofluorescence assay
IFNs: Interferons
MOI: Multiplicity of infection
Mx1: Myxovirus resistance gene
pfu: Plaque-forming unit
SCNT: Somatic cell nuclear transfer.

## References

[1] Le Bon DF (2002). Tough, Links between innate and adaptive immunity via type I interferon. Curr Opin Immunol.

[2] Haller O, Arnheiter H, Lindenmann J, Gresser I (1980). Host gene influences sensitivity to interferon action selectively for influenza virus. Nature.

[3] Haller O, Kochs G, Weber F (2007). Interferon, Mx, and viral countermeasures. Cytokine Growth Factor Rev.

[4] Pavlovic J, Haller O, Staeheli P (1992). Human and mouse Mx proteins inhibit different steps of the influenza virus multiplication cycle. J Virol.

[5] Tumpey TM, Szretter KJ, Van Hoeven N, Katz JM, Kochs G, Haller O, García-Sastre A, Staeheli P (2007). The Mx1 Gene Protects Mice against the Pandemic 1918 and Highly Lethal Human H5N1 Influenza Viruses. J Virol.

[6] Haller O, Frese M, Rost D, Nuttall PA, Kochs G (1995). Tick-borne Thogoto virus infection in mice is inhibited by the orthomyxovirus resistance gene product Mx1. J Virol.

[7] Andersson I, Bladh L, Mousavi-Jazi M, Magnusson KE, Lundkvist A, Haller O, Mirazimi A (2004). The Mx1 Gene Protects Mice against the Pandemic 1918 and Highly Lethal Human H5N1 Influenza Viruses. J Virol.

[8] Kochs G, Janzen C, Hohenberg H, Haller O (2002). Antivirally active MxA protein sequesters La Crosse virus nucleocapsid protein into perinuclear complexes. Proc Natl Acad Sci U S A.

[9] Staeheli P, Pavlovic J (1991). Inhibition of vesicular stomatitis virus mRNA synthesis by human MxA protein. J Virol.

[10] Schneider-Schaulies S, Schneider-Schaulies J, Schuster A, Bayer M, Pavlovic J, ter Meulen V (1994). Cell type-specific MxA-mediated inhibition of measles virus transcription in human brain cells. J Virol.

[11] Khaiboullina SF, Rizvanov AA, Deyde VM, St Jeor SC (2005). Andes virus stimulates interferon - inducible MxA protein expression in endothelial cells. J Med Virol.

[12] Kraus AA, Raftery MJ, Giese T, Ulrich R, Zawatzky R, Hippenstiel S, Suttorp N, Krüger DH, Schönrich G (2004). Differential antiviral response of endothelial cells after infection with pathogenic and nonpathogenic hantaviruses. J Virol.

[13] Zürcher T, Pavlovic J, Staeheli P (1992). Mechanism of human MxA protein action: variants with changed antiviral properties. EMBO J.

[14] Mundt E (2007). Human MxA protein confers resistance to double-stranded RNA viruses of two virus families. J Gen Virol.

[15] Staeheli P, Haller O, Boll W, Lindenmann J, Weissmann C (1986). Mx protein: constitutive expression in 3T3 cells transformed with cloned Mx cDNA confers selective resistance to influenza virus. Cell.

[16] Dittmann J, Stertz S, Grimm D, Steel J, García-Sastre A, Haller O, Kochs G (2008). Influenza A virus strains differ in sensitivity to the antiviral action of Mx-GTPase. J Virol.

[17] Asano A, Ko JH, Morozumi T, Hamashima N (2002). Polymorphisms and the antiviral property of porcine Mx1 protein. J Vet Med Sci.

[18] Nakajima E, Morozumi T, Tsukamoto K, Watanabe T, Plastow G, Mitsuhashi T (2007). A naturally occurring variants of porcine Mx1 associated with increased susceptibility to influenza virus in vitro. Biochem Genet.

[19] Ko JH, Jin HK, Asano A, Takada A, Ninomiya A, Kida H, Hokiyama H, Ohara M, Tsuzuki M, Nishibori M, Mizutani M, Watanabe T (2002). Polymorphisms and the differential antiviral activity of the chicken Mx gene. Genome Res.

[20] Kolb E, Laine E, Strehler D, Staeheli P (1992). Resistance to influenza virus infection of Mx transgenic mice expressing Mx protein under the control of two constitutive promoters. J Virol.

[21] Pavlovic J, Arzet HA, Hefti HP, Frese M, Rost D, Ernst B, Kolb E, Staeheli P, Haller O (1995). Enhanced virus resistance of transgenic mice expressing the human MxA protein. J Virol.

[22] Ito T, Couceiro JN, Kelm S, Baum LG, Krauss S, Castrucci MR, Donatelli I, Kida H, Paulson JC, Webster RG, Kawaoka Y (1998). Molecular basis for the generation in pigs of influenza A viruses with pandemic potential. J Virol.

[23] Smith GJ, Vijaykrishna D, Bahl J, Lycett SJ, Worobey M, Pybus OG, Ma SK, Cheung CL, Raghwani J, Bhatt S, Peiris JS, Guan Y, Rambaut A (2009). Origins and evolutionary genomics of the 2009 swine-origin H1N1 influenza A epidemic. Nature.

[24] Müller M, Brenig B, Winnacker EL, Brem G (1992). Transgenic pigs carrying cDNA copies encoding the murine Mx1 protein which confers resistance to influenza virus infection. Gene.

[25] Lai L, Kang JX, Li R, Wang J, Witt WT, Yong HY, Hao Y, Wax DM, Murphy CN, Rieke A, Samuel M, Linville ML, Korte SW, Evans RW, Starzl TE, Prather RS, Dai Y (2006). Generation of cloned transgenic pigs rich in omega-3 fatty acids. Nat Biotechnol.

[26] Yang D, Wang CE, Zhao B, Li W, Ouyang Z, Liu Z, Yang H, Fan P, O'Neill A, Gu W, Yi H, Li S, Lai L, Li XJ (2010). Expression of Huntington’s disease protein results in apoptotic neurons in the brains of cloned transgenic pigs. Hum Mol Genet.

[27] Yang D, Yang H, Li W, Zhao B, Ouyang Z, Liu Z, Zhao Y, Fan N, Song J, Tian J, Li F, Zhang J, Chang L, Pei D, Chen YE, Lai L (2011). Generation of PPARγ mono-allelic knockout pigs via zinc-finger nucleases and nuclear transfer cloning. Cell Res.

[28] Gaush CR, Smith TF (1968). Replication and plaque assay of influenza virus in an established line of canine kidney cells. Appl Microbiol.

[29] Youil R, Su Q, Toner TJ, Szymkowiak C, Kwan WS, Rubin B, Petrukhin L, Kiseleva I, Shaw AR, DiStefano D (2004). Comparative study of influenza virus replication in Vero and MDCK cell lines. J Virol Methods.

[30] Yu X, Tu C, Li H, Hu R, Chen C, Li Z, Zhang M, Yin Z (2001). DNA-mediated protection against classical swine fever virus. Vaccine.

[31] Morozumi T, Sumantri C, Nakajima E, Kobayashi E, Asano A, Oishi T, Mitsuhashi T, Watanabe T, Hamasima N (2001). Three types of polymorphisms in exon 14 in porcine Mx1 gene. Biochem Genet.

[32] Palm M, Leroy M, Thomas A, Linden A, Desmecht D (2007). Differential anti-influenza activity among allelic variants at the Sus scrofa Mx1 locus. J Interferon Cytokine Res.

[33] Donnelly ML, Luke G, Mehrotra A, Li X, Hughes LE, Gani D, Ryan MD (2001). Analysis of the aphthovirus 2A/2B polyprotein ‘cleavage’ mechanism indicates not a proteolytic reaction, but a novel translational effect: a putative ribosomal ‘skip’. J Gen Virol.

[34] Szymczak AL, Workman CJ, Wang Y, Vignali KM, Dilioglou S, Vanin EF, Vignali DA (2004). Correction of multi-gene deficiency in vivo using a single ‘self-cleaving’ 2A peptide-based retroviral vector. Nat Biotechnol.

[35] Trichas G, Begbie J, Srinivas S (2008). Use of the viral 2A peptide for bicistronic expression in transgenic mice. BMC Biol.

[36] Lee SH, Vidal SM (2002). Functional diversity of Mx proteins: variations on a theme of host resistance to infection. Genome Res.

[37] Palm M, Garigliany MM, Cornet F, Desmecht D (2010). Interferon-induced Sus scrofa Mx1 blocks endocytic traffic of incoming influenza A virus particles. Vet Res.

[38] Müller M, Winnacker EL, Brem G (1992). Molecular cloning of porcine Mx cDNAs: New members of a family of interferon-inducible proteins with homology to GTP-binding proteins. J Interferon Res.

[39] Horisberger MA (1992). Virus-specific effects of recombinant porcine interferon-gamma and the induction of Mx proteins in pig cells. J Interferon Res.

[40] Zhang X, Shin J, Molitor TW, Schook LB, Rutherford MS (1999). Molecular responses of macrophages to porcine reproductive and respiratory syndrome virus infection. Virology.

[41] Su J, Yang C, Zhu Z, Wang Y, Jang S, Liao L (2009). Enhanced grass carp reovirus resistance of Mx-transgenic rare minnow (Gobiocypris rarus). Fish Shellfish Immunol.

[42] Castrucci MR, Donatelli I, Sidoli L, Barigazzi G, Kawaoka Y, Webster RG (1993). Genetic reassortment between avian and human influenza A viruses in Italian pigs. Virology.

[43] Claas EC, Kawaoka Y, de Jong JC, Masurel N, Webster RG (1994). Infection of children with avian-human reassortant influenza virus from pigs in Europe. Virology.

[44] Kida H, Ito T, Yasuda J, Shimizu Y, Itakura C, Shortridge KF, Kawaoka Y, Webster RG (1994). Potential for transmission of avian influenza viruses to pigs. J Gen Virol.

